# Single-Cell Mapping of Genetic Risk Across Ten Respiratory Diseases

**DOI:** 10.3390/biology14121765

**Published:** 2025-12-10

**Authors:** Miao Zhou, Chao Xue

**Affiliations:** 1 Medical College, Jiaying University, Meizhou 514031, China; zhoum223@mail.sysu.edu.cn; 2 Zhongshan School of Medicine, Sun Yat-sen University, Guangzhou 510080, China; 3 Key Laboratory of Tropical Disease Control (Sun Yat-sen University), Ministry of Education, Guangzhou 510080, China

**Keywords:** single-cell transcriptomics, GWAS, respiratory diseases, genetic susceptibility, alveolar cells, immune heterogeneity, lung

## Abstract

Understanding how genetic risk variants contribute to respiratory diseases requires mapping genome-wide association study (GWAS) signals to disease-relevant cell types and states within the human lung. Here, we integrated GWAS summary statistics for ten major respiratory diseases, including asthma, COPD, idiopathic pulmonary fibrosis (IPF), COVID-19, and lung cancer, using a large-scale single-cell transcriptomic dataset of more than 523,000 cells from the Human Lung Cell Atlas. Applying the single-cell Disease Relevance Score (scDRS) framework, we systematically identified shared and disease-specific cellular associations across four major compartments, namely epithelial, immune, endothelial, and stromal. We found that alveolar type II (AT2) cells represent a central susceptibility hub for asthma, COPD, and COVID-19, whereas disease-specific risk enrichment was observed in subpopulations such as CCL3^+^ alveolar macrophages in COVID-19 and adventitial fibroblasts in asthma. Importantly, subclustering revealed substantial heterogeneity within cell types, with distinct transcriptional programs underlying differential disease associations. For example, AT2 subclusters exhibited divergent susceptibility patterns to asthma versus COVID-19, reflecting immune-interacting versus antiviral states. Our results provide a systematic single-cell framework for linking genetic risk to the cellular architecture of the human lung and uncover both shared and disease-specific mechanisms underlying respiratory disease susceptibility.

## Simple Summary

Respiratory diseases such as asthma, chronic obstructive pulmonary disease (COPD), pulmonary fibrosis, and COVID-19 affect millions of people worldwide and impose a major health burden. Although genetic studies have identified thousands of DNA variants linked to these diseases, the specific lung cell types in which these risk signals act remain unclear. In this study, we combined large-scale genetic data from ten major lung diseases with single-cell sequencing data from more than half a million human lung cells. Using a statistical method called the single-cell Disease Relevance Score (scDRS), we mapped genetic risk to specific lung cell types and even subtypes. We discovered that certain lung cells, such as alveolar type II cells, represent common targets of genetic risk for asthma, COPD, and COVID-19, whereas other cell populations, including immune cells and fibroblasts, exhibited more disease-specific effects. Importantly, we also found that not all cells of the same type are equally involved; instead, only certain subgroups within a cell type drive the genetic risk. These results provide new insights into how genetic predisposition to lung diseases is organized at the single-cell level and highlight potential cellular targets for therapy.

## 1. Introduction

Respiratory diseases, encompassing inflammatory, infectious, fibrotic, vascular, and malignant disorders, represent a major global health burden, collectively accounting for millions of deaths annually [1,2,3]. Despite substantial progress in genome-wide association studies (GWAS), which have identified numerous susceptibility loci for conditions such as asthma [4], chronic obstructive pulmonary disease (COPD) [5], idiopathic pulmonary fibrosis (IPF) [6], and lung cancer [7], the cellular contexts through which these genetic risks manifest remain poorly understood. Most GWAS signals map to noncoding regions [8], implying regulatory effects in specific cell populations rather than uniform impacts across the lung. However, linking genetic variation to disease-relevant cell types within the complex cellular ecosystem of the human lung remains a major challenge.

Single-cell RNA sequencing (scRNA-seq) has revolutionized our understanding of lung biology by enabling high-resolution mapping of cellular diversity and state-specific transcriptional programs [9,10]. In recent years, multiple integrative methods have been developed to link GWAS summary statistics with scRNA-seq data, allowing the mapping of genetic risk to specific cell types [11,12] and even individual cells [13], and enabling the exploration of cellular heterogeneity underlying complex traits. However, a systematic analysis of disease-associated cell types across the full spectrum of respiratory diseases and an in-depth investigation of the within-cell-type heterogeneity that contributes to differential susceptibility remain lacking.

To address these gaps, we integrated GWAS summary statistics from ten major respiratory diseases with a single-cell atlas of over 523 K human lung cells using the single-cell Disease Relevance Score (scDRS) [13]. This analysis systematically mapped genetic risk across endothelial, epithelial, immune, and stromal compartments, revealing both shared and disease-specific cellular associations as well as substantial heterogeneity within key cell types. Our study provides a comprehensive cellular framework of genetic susceptibility for respiratory diseases and highlights potential targets for precision therapies.

## 2. Materials and Methods

### 2.1. Overall Analytical Strategy

We performed a comprehensive analysis to investigate how genetic risk for respiratory diseases maps onto the cellular composition of the human lung. Specifically, we integrated publicly available GWAS summary statistics from ten respiratory diseases with a large-scale single-cell transcriptomic dataset comprising approximately 523 K human lung cells.

To link genetic risk to individual cells, we adopted the single-cell Disease Relevance Score (scDRS) method [13], a statistical framework designed to evaluate the enrichment of disease-associated genes at single-cell resolution. This method utilizes GWAS-derived gene-level scores and incorporates both gene expression levels and cellular variability to assign each cell a disease relevance score. scDRS thus allows for a quantitative assessment of how strongly a cell’s transcriptional profile aligns with disease-associated genetic signals.

Our analysis followed a two-step workflow:

**(a) Cell type and disease association mapping**. We first systematically assessed the association between disease genetic risk and predefined lung cell types. The cells were categorized into four broad compartments: immune, epithelial, endothelial, and stromal, based on canonical marker gene expression. For each disease, we evaluated whether specific cell types were significantly enriched for disease risk using aggregated scDRS scores. We also tested for intra-cell type heterogeneity, assessing whether risk signals were evenly distributed across cells of the same type.

**(b) Analysis of heterogeneity within associated cell types**. For disease and cell type pairs showing significant heterogeneity, we conducted detailed investigations to identify the specific subpopulations or subclusters driving the observed risk enrichment. This included subcluster-level scDRS evaluation and functional characterization of the corresponding marker genes to infer the potential biological roles of risk-associated subtypes.

In this study, we focus on applying an existing method in a systematic and integrative way to comprehensively map the genetic risk landscape of multiple respiratory diseases at single-cell resolution. By doing so, we aim to better understand both shared and disease-specific cellular mechanisms underlying genetic susceptibility.

### 2.2. GWAS Summary Statistics Collection

We collected GWAS summary statistics for nine respiratory diseases from the GWAS Catalog database (https://www.ebi.ac.uk/gwas/ (accessed on 3 July 2025)) and COVID-19 data from the Host Genetics Initiative (HGI, Release 7, downloaded from https://www.covid19hg.org/results/r7/ (accessed on 3 July 2025)) (Table 1). The selected diseases encompass a wide range of pulmonary conditions with distinct etiologies and clinical manifestations, including: Asthma [4], Chronic Obstructive Pulmonary Disease (COPD) [5], COVID-19 [14], Idiopathic Fibrosing Alveolitis (IFA) [15], Idiopathic Pulmonary Fibrosis (IPF) [6], Influenza Susceptibility (FLU) [16], Lung Adenocarcinoma (LUAD) [17], Non-Small Cell Lung Cancer (NSCLC) [7], Pulmonary Arterial Hypertension (PAH) [18], Tuberculosis (TB) [15]. All datasets were obtained in the form of publicly available GWAS summary statistics, including single-nucleotide polymorphism (SNP) effect sizes, standard errors, *p*-values, and allele frequency information where available.

The majority of the included GWAS studies were conducted in European-ancestry cohorts, which we accounted for in downstream linkage disequilibrium (LD) computations. Specifically, we used Phase 3 of the 1000 Genomes Project (European subset) [19] as the LD reference panel for relevant calculations. The sample sizes of the GWAS datasets varied across diseases, with an average total sample size of approximately 599 K individuals. This large-scale genetic association data provided the foundation for subsequent integration with single-cell expression profiles to assess the distribution of genetic risk across different lung cell types.

### 2.3. Human Lung Single-Cell Transcriptomic Data

We used a large-scale human lung single-cell RNA sequencing dataset from the Human Lung Cell Atlas (HLCA) (https://data.humancellatlas.org/hca-bio-networks/lung/atlases/lung-v1-0 (accessed on 4 July 2025)) [20]. The dataset comprises approximately collected from 107 healthy individuals, covering multiple anatomical sites including the lung parenchyma, respiratory airway, and nasal tissue. For downstream analysis, we used the pre-integrated and batch-corrected gene expression matrices provided by HLCA. The data underwent prior processing steps, including quality control, batch effect correction across individuals and platforms. Cell clustering and annotation were performed by the HLCA consortium. Each cell was assigned a type based on the “ann_finest_level” annotation, representing the finest labeling system in the dataset. In total, 61 distinct cell types were defined. These cell types were further grouped into four broad categories, namely immune, epithelial, endothelial, and stromal cells, based on the annotations provided by HLCA.

### 2.4. Inference of Disease-Associated Cells

To infer disease-associated cells at single-cell resolution, we applied the scDRS method [13]. scDRS integrates genome-wide association study (GWAS) signals with single-cell transcriptomic data by calculating a multi-gene score for each cell, thereby assessing how strongly a cell’s expression profile reflects genetic predisposition to disease.

We implemented scDRS using the publicly available code from the official repository (https://github.com/martinjzhang/scDRS (accessed on 5 July 2025)), version v1.0.2. All analyses were performed following the recommended pipeline, and non-essential parameters were kept at default settings. As required by the scDRS pipeline, we first performed gene-level association analysis using MAGMA [21] (v1.10) [https://cncr.nl/research/magma (accessed on 5 July 2025)]. MAGMA aggregates SNP-level GWAS summary statistics into gene-level Z-scores, correcting for factors such as gene size, local linkage disequilibrium, and SNP density. These gene-level Z-scores serve as input to scDRS, which then calculates a disease relevance score for each individual cell. The scoring procedure assesses whether a cell shows significant overexpression of disease-associated genes compared to randomly sampled control gene sets. In our analysis, both the disease gene set and the matched control gene sets were set to a size of 1000 genes. Single cells were considered significantly associated with the disease if their Benjamini–Hochberg false discovery rate (FDR) was <0.1.

### 2.5. Cell-Type Level Association and Heterogeneity Analysis

We quantified disease associations at the cell-type level using the scDRS. For each disease–cell type pair, we calculated the top 5% quantile of the cell-level disease relevance scores as the test statistic, following the default scDRS implementation. This approach evaluates whether a specific cell type is significantly enriched for high-scoring cells, while being robust to potential outliers or annotation errors.

To evaluate within-cell-type heterogeneity, scDRS applied Geary’s C statistic, a spatial autocorrelation measure adapted for single-cell data. Geary’s C tests whether cells with similar disease relevance scores tend to cluster in the cell–cell similarity network based on transcriptomic profiles. A significantly low Geary’s C value indicates the presence of subpopulations within the same cell type that disproportionately contribute to the disease association, reflecting heterogeneous genetic risk enrichment.

Both association and heterogeneity tests were corrected for multiple comparisons using the FDR procedure, with results considered significant at FDR < 0.05.

### 2.6. Analysis of Disease-Associated Cell Subtype Heterogeneity

For cell types exhibiting significant within-cell-type heterogeneity in disease association, we performed sub-clustering analysis to identify potential disease-relevant subpopulations. Specifically, we extracted all cells belonging to the heterogeneous cell type and re-clustered them using the Leiden algorithm implemented in the Scanpy package [22] (version 1.9.3). We selected the top 2000 highly variable genes as input features for clustering, constructed the neighborhood graph with n_neighbors = 10 and performed Leiden clustering with resolution = 0.5 to control cluster granularity.

After initial clustering, we examined the distribution of disease relevance scores across the resulting subclusters. If multiple clusters showed consistent disease association patterns, we merged them to reduce redundancy and maintain interpretability.

To characterize the functional differences among these cell subtypes, we identified cluster-specific marker genes using the Wilcoxon rank-sum test as implemented in Scanpy’s “rank_genes_groups” function. The top 200 marker genes for each subcluster were selected for downstream functional enrichment analysis. We also tested using the top 100, 300, and 500 marker genes to evaluate the robustness of the enrichment results. Enrichment analyses were conducted using g:Profiler [23] (https://biit.cs.ut.ee/gprofiler/gost (accessed on 15 July 2025)), focusing on the Kyoto Encyclopedia of Genes and Genomes (KEGG) [24] and Gene Ontology [25]. Biological Process (GO:BP) databases. This allowed us to infer the potential biological roles of disease-associated subclusters. Adjusted *p* values were calculated using the multiple testing correction method provided by g:Profiler, and terms with adjusted *p* < 0.05 were considered significant.

### 2.7. Code Availability

The complete analysis pipeline, including data preprocessing, statistical analysis, and figure generation, is available at: https://github.com/chaoxue-gwas/LungDiseaseCell (accessed on 27 November 2025).

## 3. Results

**Figure 1:**
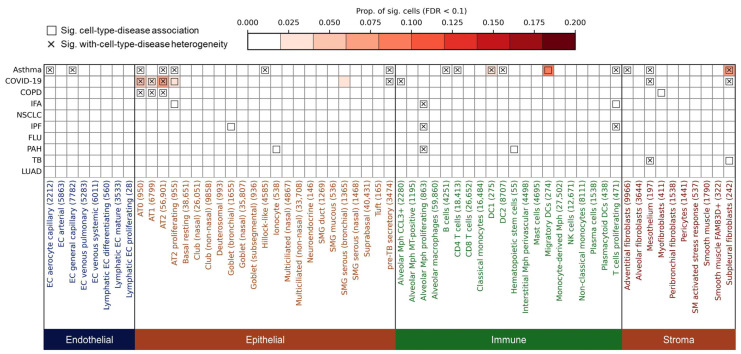
Cell type-level associations and heterogeneity between respiratory diseases and human lung cells. The heatmap shows the association results between ten respiratory diseases (y-axis; disease abbreviations are defined in Table 1) and lung cell types (x-axis). Cell type labels are followed by the number of constituent cells in parentheses. Colors indicate the major cell category: endothelial, epithelial, immune, or stromal. The color intensity represents the proportion of significantly associated cells within each cell type (FDR < 0.1). Boxes indicate significant disease–cell type associations at the cell-type level (FDR < 0.05). Cross marks (×) denote significant heterogeneity of disease association within the cell type (FDR < 0.05), indicating that disease-relevant scores are unevenly distributed across individual cells in that cell type. Complete data used to generate this figure are provided in Supplementary Table S1.

To investigate how genetic risk for respiratory diseases maps onto the cellular composition of the human lung, we performed a comprehensive analysis. We collected summary statistics of GWAS for ten respiratory diseases from public databases (Table 1), covering five categories of respiratory diseases with different etiologies: inflammatory (asthma, COPD), infectious (COVID-19, FLU, TB), fibrotic (IPF, IFA), vascular (PAH), and malignant (NSCLC, LUAD) disorders. We then integrated these GWAS data with a large-scale single-cell transcriptome dataset of approximately 523 K human lung cells. To identify shared and disease-specific genetically associated cell types, we systematically analyzed associations across four major cell compartments: endothelial, epithelial, immune, and stromal cells (Figure 1).

### 3.1. Overview of Respiratory Disease Associations and Heterogeneity Across Lung Cell Types

Among endothelial cells (n = 31,272, eight subtypes), significant associations were observed exclusively for asthma, involving two capillary subtypes: endothelial cell (EC) aerocyte capillary (FDR = 0.047) and EC general capillary (gCap) (FDR = 0.047), while no other diseases showed endothelial enrichment (Figure 1; all data underlying Figure 1 are available in Supplementary Table S1). This suggests a disease-specific endothelial vulnerability in asthma. Aerocytes and gCap cells are both involved in vasomotor control, lipid metabolism, and hemostasis. In a mouse model of alveolar injury and emphysema induced by elastase, labeled gCap cells were observed to proliferate significantly three days after injury, indicating that they play a key role in lung repair. In contrast, air cells did not migrate or proliferate after injury, suggesting that they may play a role in fixation or quiescence [26].

Within the epithelial compartment (n = 272,108, 23 subtypes), several alveolar lineages emerged as shared susceptibility cell types. Both Alveolar Type (AT) 0 and AT2 cells were significantly associated with asthma (FDR_AT0_ = 0.020, FDR_AT2_ = 0.035), COPD (FDR_AT0_ = 0.020, FDR_AT2_ = 0.020), and COVID-19 (FDR_AT0_ = 0.020, FDR_AT2_ = 0.020), while AT1 cells were linked to COVID-19 (FDR *=* 0.020) and COPD (FDR *=* 0.020), and AT2 proliferating cells to asthma (FDR = 0.020), COVID-19 (FDR *=* 0.020), and IFA (FDR = 0.035) (Figure 1). These findings are consistent with prior evidence that AT2 cells serve as primary targets of SARS-CoV-2 infection and are implicated in epithelial regeneration and inflammation in COPD and asthma [27,28,29,30]. Other epithelial subtypes showed more disease-specific patterns: Pre-TB secretory cells were associated with asthma (FDR = 0.020) and COVID-19 (FDR = 0.047), Goblet cells with IPF (FDR = 0.047), and Hillock-like epithelial cells specifically with asthma (FDR = 0.035), possibly reflecting epithelial hyperplasia and mucin production in airway remodeling [31,32,33] (Figure 1).

The immune compartment (n = 199,233, 20 subtypes) revealed disease-specific enrichment for distinct myeloid and lymphoid populations. Alveolar macrophage (Mph) CCL3+ cells were associated with COVID-19 (FDR = 0.020, Figure 1), in line with pro-inflammatory monocyte-derived alveolar infiltration during acute infection [34]. In contrast, Alveolar Mph proliferating cells were linked to IFA (FDR = 0.035), IPF (FDR = 0.020), and PAH (FDR = 0.020), suggesting a role in chronic fibrotic inflammation (Figure 1). Several adaptive immune populations, including B cells (FDR = 0.020), CD4 T cells (FDR = 0.020), DC1 (FDR = 0.020), DC2 (FDR = 0.020), migratory DCs (FDR = 0.020), and proliferating T cells (FDR = 0.020), were specifically associated with asthma (Figure 1), supporting the contribution of adaptive immunity to disease pathogenesis [35,36,37,38]. Notably, T cell proliferating cells were also enriched in IFA and IPF (*P*_IFA_ = 0.020, *P*_IPF_ = 0.020), highlighting shared inflammatory features among fibrotic and allergic diseases (Figure 1).

Finally, in the stromal compartment (n = 20,088, 10 subtypes), we observed disease-specific associations across fibroblast subsets. Adventitial fibroblasts were uniquely associated with asthma (FDR = 0.020, Figure 1), while myofibroblasts showed enrichment in COPD (FDR = 0.020, Figure 1), consistent with airway wall thickening and fibrosis [39,40,41]. Mesothelial cells and subpleural fibroblasts were associated with asthma, COVID-19, and TB (FDR = 0.020, Figure 1), suggesting convergent mesenchymal responses to both inflammatory and infectious stimuli.

These findings delineate a landscape of both shared genetically associated cell types—such as AT2 cells in asthma, COPD, and COVID-19—and disease-specific susceptibility cells, shedding light on the cellular basis of respiratory disease heterogeneity.

To further assess relationships among diseases, we constructed a correlation matrix based on cell-type association scores (Supplementary Figure S1). Among all trait pairs, IFA and IPA showed the strongest correlation (Spearman’s r = 0.60), indicating that these two conditions share substantial similarity in their underlying disease-associated cellular signatures. In contrast, COVID-19 and asthma exhibited a moderate correlation (r = 0.46), suggesting partial but less extensive overlap in the cellular programs implicated by these diseases.

We observed that most disease–cell type associations exhibited significant heterogeneity (Figure 1). For instance, within endothelial cells, asthma showed heterogeneous associations with EC aerocyte capillary (FDR = 0.010) and EC general capillary (FDR = 0.016, Figure 1). In epithelial cells, AT0 cells displayed heterogeneous associations with asthma, COVID-19, and COPD (FDR = 0.010, Figure 1); AT1 cells exhibited heterogeneity in its associations with COVID-19 and COPD (FDR_COVID-19_ = 0.016, FDR_COPD_ = 0.030, Figure 1); AT2 cells showed heterogeneous associations with asthma, COVID-19, and COPD (FDR_asthma_ = 0.010, FDR_COVID-19_ = 0.016, FDR_COPD_ = 0.030, Figure 1). Moreover, hillock-like cells demonstrated heterogeneity in their association with asthma (FDR = 0.030, Figure 1), while pre-TB secretory cells displayed heterogeneity in their associations with both asthma and COVID-19 (FDR_asthma_ = 0.010, FDR_COVID-19_ = 0.033, Figure 1). Among immune cells, alveolar macrophages (Mph) CCL3+ showed heterogeneity in their association with COVID-19 (FDR = 0.010), whereas proliferating alveolar macrophages exhibited heterogeneous associations with IFA, IPF, and PAH (FDR_IFA_ = 0.020, FDR_IPF_ = 0.047, FDR_PAH_ = 0.034, Figure 1). Asthma also demonstrated heterogeneous associations with B cells (FDR = 0.010), CD4 T cells (FDR = 0.010), DC1 (FDR = 0.010), DC2 (FDR = 0.010), and proliferating T cells (FDR = 0.025, Figure 1), with the latter additionally showing heterogeneity in its association with IPF (FDR = 0.016, Figure 1). Within stromal cells, adventitial fibroblasts exhibited heterogeneity in their association with asthma (FDR = 0.010, Figure 1), while mesothelium displayed heterogeneous associations with asthma, COVID-19, and TB (FDR_asthma_ = 0.010, FDR_COVID-19_ = 0.010, FDR_TB_ = 0.016, Figure 1). Similarly, subpleural fibroblasts showed heterogeneous associations with asthma, COVID-19, and TB (FDR_asthma_ = 0.010, FDR_COVID-19_ = 0.010, Figure 1).

### 3.2. Heterogeneous Genetic Associations of AT2 Cell Subpopulations with Asthma, COVID-19, and COPD

**Figure 2:**
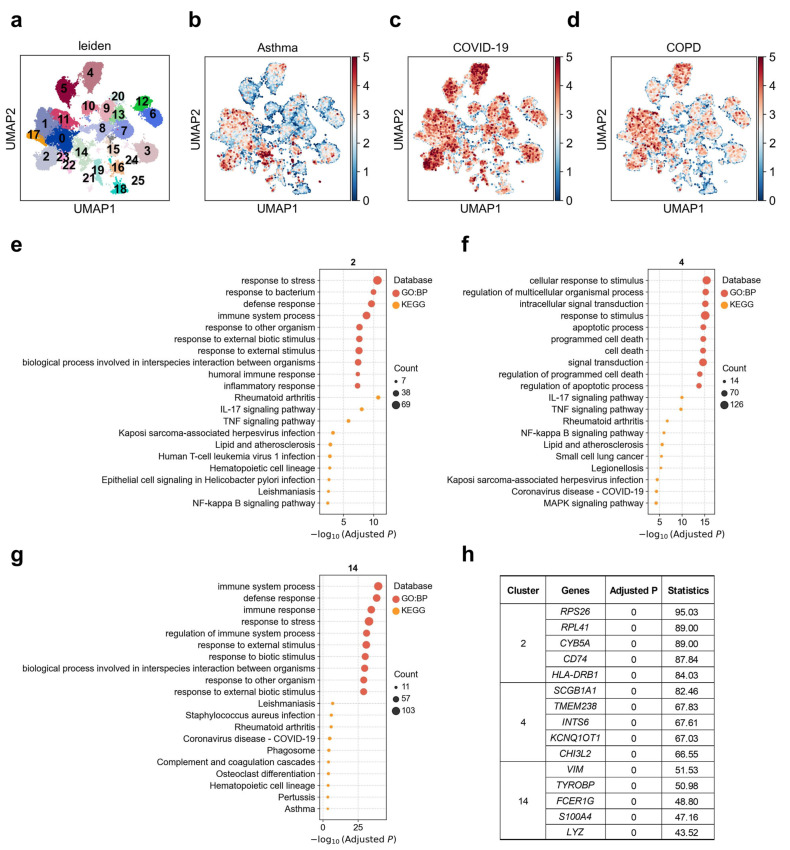
Heterogeneity of alveolar type II (AT2) cells associations with asthma, COVID-19, and COPD. (**a**) Re-clustering of AT2 cells using the Leiden algorithm. Each dot represents a single cell, displayed in two-dimensional UMAP space. Colors indicate different Leiden clusters, with numeric labels corresponding to cluster IDs. (**b**–**d**) Disease relevance scores of AT2 cells for (**b**) asthma, (**c**) COVID-19, and (**d**) COPD. Higher values indicate stronger disease associations. Axes are consistent with panel (**a**). (**e**–**g**) Functional enrichment analysis of the top 200 marker genes from (**e**) cluster 2, (**f**) cluster 4, and (**g**) cluster 14. The y-axis lists enriched functional terms, while the x-axis shows the negative log10 of the adjusted *p* values. Dot color denotes the annotation source (e.g., KEGG or GO: BP), and dot size represents the number of overlapping genes between the cluster marker set and each term. (**h**) The top 5 marker genes for each AT2 cluster. “Adjusted *p*” refers to *p* values from the Wilcoxon rank-sum test, corrected using the Benjamini–Hochberg FDR method. “Statistics” denotes the corresponding standardized test statistic (z-score) from the Wilcoxon rank-sum test.

For a long time, it has been believed that AT2 cells in the alveoli play the role of progenitor cells. Once AT1 cells are damaged, neighboring AT2 cells will be stimulated to proliferate and transdifferentiate into AT1 cells [42]. To dissect disease-associated cellular heterogeneity within alveolar type II (AT2) cells, we performed subclustering and identified 25 transcriptionally distinct AT2 subpopulations. Among these, cluster 14 exhibited a strong and specific association with asthma compared to other clusters (Figure 2b), with 6.8% of cells in this subcluster significantly associated (FDR < 0.1) with asthma, whereas only 0.3% were associated with COPD (Figure 2d; Supplementary Table S2). For COVID-19, clusters 2 and 4 displayed the highest genetic risk enrichment, with 16.2% and 18.4% of cells in these subclusters significantly associated with COVID-19, respectively. COPD showed strong associations with clusters 0, 1, 4, and 11. To further investigate the functional diversity of these disease-associated AT2 subpopulations, we conducted GO and KEGG enrichment analyses on the top 200 upregulated genes from clusters 2, 4, and 14 (Figure 2e–g). Cluster 2 was significantly enriched for pathways involved in innate immunity and microbial response, such as “response to bacterium” (adjusted *p* = 1.07 × 10^−10^), “humoral immune response” (adjusted *p* = 4.23 × 10^−8^), “inflammatory response” (adjusted *p* = 4.63 × 10^−8^), and signaling pathways including *TNF*, *IL-17*, and *NF-κB* (Figure 2e). To ensure robustness, we repeated the functional enrichment analysis using different cut-offs of top upregulated genes (top 100, 200, 300 and 500); all yielded broadly consistent pathway signatures (Supplementary Table S3). Notably, this cluster was also enriched for genes implicated in “Kaposi sarcoma-associated herpesvirus infection” (adjusted *p* = 0.0005), “Helicobacter pylori infection” (adjusted *p* = 0.002), and “leishmaniasis” (adjusted *p* = 0.003) (Figure 2e). Key upregulated genes in cluster 2 included *RPS26*, *RPL41*, *CYB5A*, *CD74*, and *HLA-DRB1* (Figure 2h). Among these, *CD74* and *HLA-DRB1* are well-established immune-related genes, with *CD74* serving as the invariant chain of MHC class II molecules and playing a crucial role in antigen presentation [43], while *HLA-DRB1* is a key component of the human leukocyte antigen system, essential for adaptive immunity [44]. Cluster 4 demonstrated enrichment for pathways associated with programmed cell death, apoptosis, and signal transduction, such as “regulation of apoptotic process” (adjusted *p* = 2.06 × 10^−15^), “MAPK signaling” (adjusted *p* = 5.74 × 10^−5^), and *TNF* (adjusted *p* = 1.81 × 10^−10^) and *IL-17* signaling (adjusted *p* = 1.12 × 10^−10^). Additionally, this cluster was significantly associated with pathways related to COVID-19, small cell lung cancer, and *NF-κB* activation (Figure 2f). Key upregulated genes in cluster 4 included *SCGB1A1*, *TMEM238*, *INTS6*, *KCNQ1OT1*, and *CHI3L2* (Figure 2h). Notably, *CHI3L2* belongs to the chitinase-like protein family, which has been implicated in inflammatory responses and tissue remodeling, with recent study highlighting its upregulation in the context of viral infections and lung inflammation [45]. The top upregulated genes in cluster 14, namely, *VIM*, *TYROBP*, *FCER1G*, *LYZ*, and *S100A4*, in contrast, was selectively enriched for asthma-related pathways, including “immune response” (adjusted *p* = 5.94 × 10^−35^), “defense response” (adjusted *p* = 8.97 × 10^−39^), and “regulation of immune system process” (adjusted *p* = 1.23 × 10^−31^) (Figure 2g,h).

### 3.3. Heterogeneous Genetic Associations of Hillock-like Cell Subpopulations with Asthma

**Figure 3:**
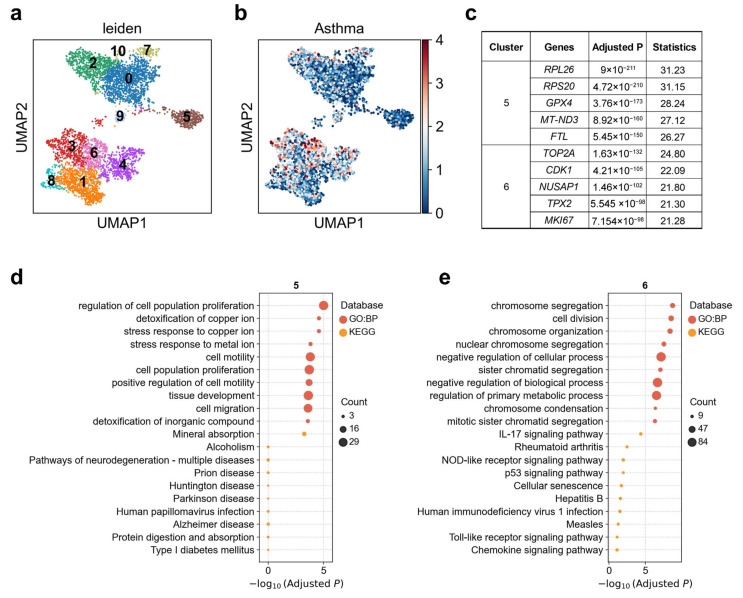
Heterogeneity of hillock-like cell associations with asthma. (**a**) Re-clustering of hillock-like cells using the Leiden algorithm. Each dot represents a single cell, displayed in two-dimensional UMAP space. Colors indicate different Leiden clusters, with numeric labels corresponding to cluster IDs. (**b**) Disease relevance scores of hillock-like cells for asthma. Axes are consistent with panel (**a**). (**c**) The top 5 marker genes for each cluster of hillock-like cells. “Adjusted *p*” refers to *p* values from the Wilcoxon rank-sum test, corrected using the Benjamini–Hochberg FDR method. “Statistics” denotes the corresponding standardized test statistic (z-score) from the Wilcoxon rank-sum test. (**d**,**e**) Functional enrichment analysis of the top 200 marker genes from (**d**) cluster 5, and (**e**) cluster 6. The y-axis lists enriched functional terms, while the x-axis shows the negative log10 of the adjusted *p* values. Dot color denotes the annotation source, and dot size represents the number of overlapping genes between the cluster marker set and each term.

Airway hillocks are stratified epithelial structures of unknown function Hillocks persist for months and harbor a unique population of basal stem cells that express genes associated with barrier function and cell adhesion. Hillock basal stem cells continually replenish overlying squamous barrier cells [32]. We found that the association between hillock-like cells and asthma exhibits clear heterogeneity. Upon reclustering these hillock-like cells into 11 subclusters (Figure 3a), asthma exhibited a markedly stronger association with cluster 6, with 4.0% of cells in this subcluster significantly associated, whereas the weakest association was observed with cluster 5, with 0% of cells significantly associated (Figure 3b; Supplementary Table S2). Cluster 5 demonstrated upregulation of *RPL/RPSs*, *GPX4*, *FTL*, *MT-ND3*, along with genes related to ribosomal and chromatin regulation [46] (Figure 3c). This cluster was enriched for pathways such as metal ion stress/copper detoxification, regulation of cell population proliferation (*p* < 0.0002), and tissue development (*p* = 0.0002), collectively suggesting a buffering state characterized by antioxidant-metal homeostasis and mitochondrial metabolic adaptation [47] (Figure 3d). Notably, *GPX4* plays a central role in protecting cells against oxidative damage and ferroptosis by reducing membrane phospholipid peroxides—an important mechanism implicated in asthma pathogenesis [48]. Cluster 6 was enriched for G_2_/M-phase mitotic genes such as *MKI67*, *TOP2A*, *CDK1*, *TPX2*, *NUSAP1*, accompanied by upregulation of inflammation-innate immunity pathways, including IL-17 signaling, NOD-like receptor signaling, and Toll-like receptor signaling [49,50] (Figure 3c,e). This expression profile is characteristic of highly proliferative, repair-oriented cell states, consistent with a regenerative response following injury [51,52]. *MKI67*, *TOP2A*, and *CDK1* are canonical markers of active cell cycling and proliferation [50,53]. These two subclusters plausibly represent the dual functional poles of the hillock cell unit: a damage-resistant buffering end (Cluster 5) and a repair-oriented regenerative end (Cluster 6). This dichotomy aligns with the gradient of asthma-related genetic association observed—cluster 5 having the lowest, and cluster 6 the highest, asthma-related scDRS scores.

### 3.4. Heterogeneous Genetic Associations of Pre-TB Secretory Cell Subpopulations with Asthma and COVID-19

**Figure 4:**
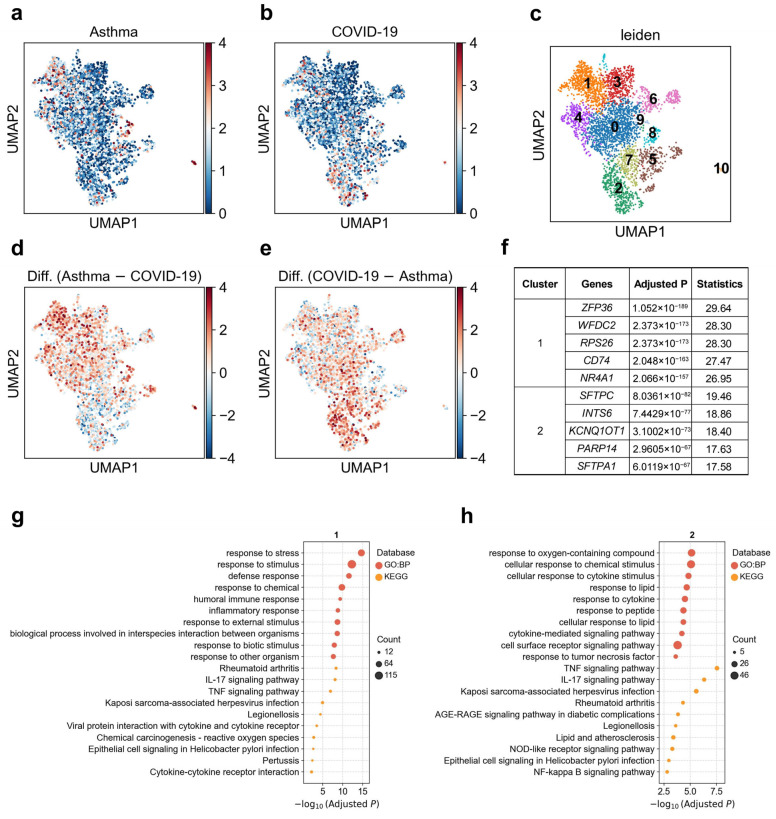
Heterogeneity of pre-TB secretory cell associations with asthma and COVID-19. (**a**,**b**) Disease relevance scores of pre-TB secretory cells for (**a**) asthma and (**b**) COVID-19. Higher values indicate stronger disease associations. Each dot represents a single cell, displayed in two-dimensional UMAP space. (**c**) Re-clustering of pre-TB secretory cells using the Leiden algorithm. Colors indicate different Leiden clusters, with numeric labels corresponding to cluster IDs. (**d**) Difference in disease relevance scores between asthma and COVID-19 (asthma minus COVID-19). Positive values indicate cells more strongly associated with asthma than COVID-19. (**e**) Difference in disease relevance scores between COVID-19 and asthma (COVID-19 minus asthma). Positive values indicate cells more strongly associated with COVID-19 than asthma. (**f**) The top 5 marker genes for each cluster of pre-TB secretory cells. “Adjusted *p*” refers to *p* values from the Wilcoxon rank-sum test, corrected using the Benjamini–Hochberg FDR method. “Statistics” denotes the corresponding standardized test statistic (z-score) from the Wilcoxon rank-sum test. (**g**,**h**) Functional enrichment analysis of the top 200 marker genes from (**g**) cluster 1, and (**h**) cluster 2. The y-axis lists enriched functional terms, while the x-axis shows the negative log10 of the adjusted *p* values. Dot color denotes the annotation source, and dot size represents the number of overlapping genes between the cluster marker set and each term.

Remodeling and loss of distal conducting airways, including preterminal and terminal bronchioles (pre-TBs/TBs), underlie progressive airflow limitation [54]. We observed marked heterogeneity in the associations between pre-TB secretory cells and asthma versus COVID-19 (Figure 4a,b). When we reclustered this cell population (Figure 4c), we identified two major subclusters that differed in their disease associations. Cluster 1 showed a stronger association with asthma (1.6% significantly associated cells), whereas cluster 2 was more strongly associated with COVID-19 (5.8% significantly associated cells, Figure 4d,e; Supplementary Table S2). Although both clusters were enriched for similar immune-related pathways such as TNF, IL-17, and cytokine–receptor signaling (Figure 4g,h), their top marker genes were distinct. Cluster 1 displayed elevated expressions of *ZFP36*, *WFDC2*, *RPS26*, *CD74*, and *NR4A1*, while cluster 2 was characterized by higher expressions of *SFTPC*, *SFTPA1*, *INTS6*, *KCNQ1OT1*, and *PARP14* (Figure 4f). These results suggest that pathway-level enrichment alone may not sufficiently distinguish between the two subclusters, whereas their distinct marker gene expression profiles reveal different cellular identities and explain their divergent disease associations.

### 3.5. Heterogeneous Genetic Associations of Alveolar CCL3^+^ Macrophage Subpopulations with COVID-19

**Figure 5:**
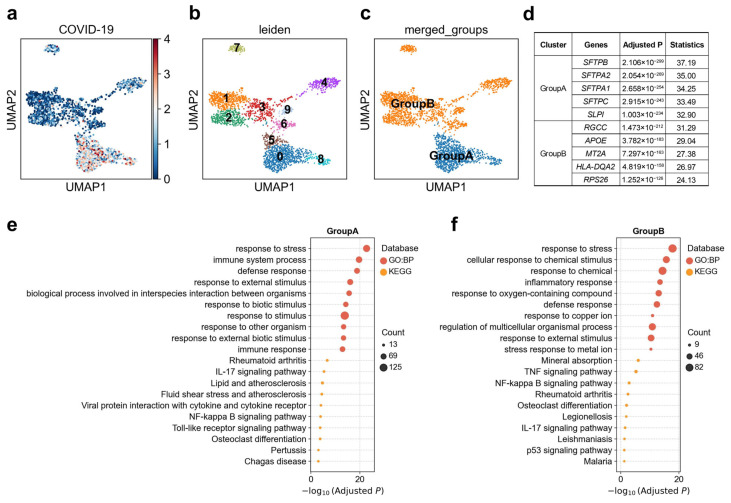
Heterogeneity of alveolar Mph CCL3+ cell associations with COVID-19. (**a**) Disease relevance scores of alveolar Mph CCL3+ cells for COVID-19. Higher values indicate stronger disease associations. Each dot represents a single cell, displayed in two-dimensional UMAP space. (**b**) Re-clustering of alveolar Mph CCL3+ cells using the Leiden algorithm. Colors indicate different Leiden clusters, with numeric labels corresponding to cluster IDs. (**c**) Based on the disease score distribution patterns, clusters 0, 5, and 8 were merged into GroupA, and the remaining clusters were merged into GroupB. (**d**) The top 5 marker genes for each cluster of alveolar Mph CCL3+ cells. “Adjusted *p*” refers to *p* values from the Wilcoxon rank-sum test, corrected using the Benjamini–Hochberg FDR method. “Statistics” denotes the corresponding standardized test statistic (z-score) from the Wilcoxon rank-sum test. (**e**,**f**) Functional enrichment analysis of the top 200 marker genes from cell subpopulation (**e**) GroupA, and (**f**) GroupB. The y-axis lists enriched functional terms, while the x-axis shows the negative log10 of the adjusted *p* values. Dot color denotes the annotation source, and dot size represents the number of overlapping genes between the cluster marker set and each term.

Initial phagocytosis by alveolar macrophages leads to the production of proinflammatory cytokines and chemokines [55]. We observed pronounced cellular heterogeneity in the association between alveolar Mph CCL3^+^ cells and COVID-19 (Figure 5a). By reclustering this population into ten distinct subclusters and examining single-cell-level disease association patterns, we identified significant differences in disease-related association strength between individual clusters (Figure 5b). Subsequent analysis resolved two major functional groups within these reclustered Mph CCL3^+^ cells (Figure 5c): Group A (clusters 0, 5, and 8) exhibited marked upregulation of *SFTPB*, *SFTPA2*, *SFTPA1*, *SFTPC*, and *SLPI*, and was enriched for immune system process such as “IL-17 signaling pathway” and “NF-kappa B signaling pathway”, and response to stress especially for organisms; Group B, by contrast, was characterized by elevated expression of *RGCC*, *APOE*, *MT2A*, *HLA-DQA2*, and *RPS26*, among other genes associated with immune system process such as “TNF signaling pathway”, “IL-17 signaling pathway” and “NF-kappa B signaling pathway”, and cellular response to chemical stimulus (Figure 5d–f). In our scDRS analysis, Group A displayed significantly stronger genetic association with COVID-19 than Group B. This suggests that distinct functional states of macrophages may differentially mediate the localization of infection-related genetic risk at the single-cell level.

### 3.6. Heterogeneous Genetic Associations of Adventitial Fibroblast Subpopulations with Asthma

**Figure 6:**
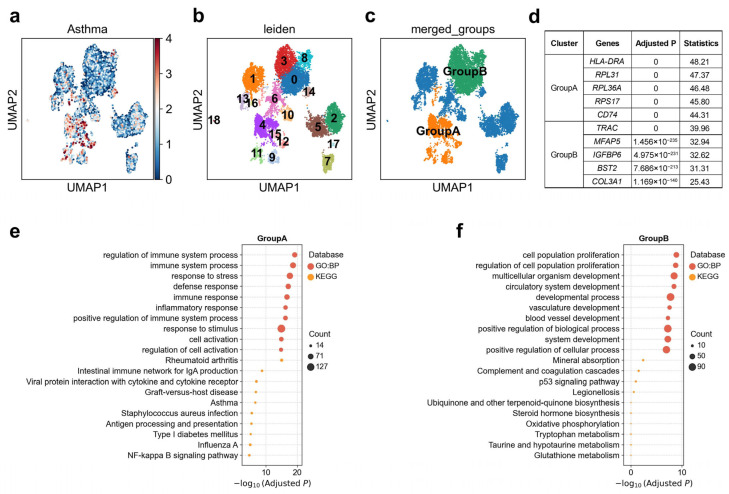
Heterogeneity of adventitial fibroblasts associations with asthma. (**a**) Disease relevance scores of adventitial fibroblasts for asthma. Higher values indicate stronger disease associations. Each dot represents a single cell, displayed in two-dimensional UMAP space. (**b**) Re-clustering of adventitial fibroblasts using the Leiden algorithm. Colors indicate different Leiden clusters, with numeric labels corresponding to cluster IDs. (**c**) Based on the disease score distribution patterns, clusters 4, 9, 11, 12, and 15 were merged into GroupA, and clusters 0, 3, 8 were merged into GroupB. (**d**) The top 5 marker genes for each cluster of adventitial fibroblasts. “Adjusted *p*” refers to *p* values from the Wilcoxon rank-sum test, corrected using the Benjamini–Hochberg FDR method. “Statistics” denotes the corresponding standardized test statistic (z-score) from the Wilcoxon rank-sum test. (**e**,**f**) Functional enrichment analysis of the top 200 marker genes from cell subpopulation (**e**) GroupA, and (**f**) GroupB. The y-axis lists enriched functional terms, while the x-axis shows the negative log10 of the adjusted *p* values. Dot color denotes the annotation source, and dot size represents the number of overlapping genes between the cluster marker set and each term.

Pulmonary artery adventitial fibroblasts were shown to direct the behavior of immune cells and serving as a homing place for the immune cells during vascular remodeling [56]. We observed substantial heterogeneity in the genetic association between adventitial fibroblasts and asthma (Figure 6a). Reclustering analysis further resolved this cell type into 19 transcriptionally distinct clusters (Figure 6b), revealing marked differences in disease association strength across subpopulations. Based on single-cell-level association patterns, adventitial fibroblasts could be stratified into two major functional groups (Figure 6c). Group A (clusters 4/9/11/12/15) was characterized by strong expression of *HLA-DRA*, *CD74*, and was enriched for pathways related to antigen processing and presentation, NF-κB signaling, and inflammatory responses (Figure 6d,e), with 2.1% of cells in these clusters significantly associated with the disease (Supplementary Table S2). In contrast, Group B (clusters 0/3/8) was enriched for extracellular-matrix and vascular/metabolic genes, including *MFAP5*, and *COL3A1*, with pathway enrichment for vascular development, oxidative phosphorylation, and glutathione metabolism (Figure 6d,f), with only 0.06% of cells in these clusters significantly associated with the disease (Supplementary Table S2). This expression profile suggests a tissue-remodeling/metabolic-adaptation state, consistent with fibroblast contributions to extracellular matrix turnover and airway structural changes in chronic asthma [57]. Notably, Group A exhibited significantly stronger genetic association with asthma in scDRS analysis than Group B, indicating that asthma risk variants are preferentially localized to fibroblast subpopulations with an immune-activated, antigen-presenting phenotype. This highlights the dual roles of fibroblasts in asthma pathogenesis: not only as structural mediators of remodeling but also as active participants in immune regulation.

## 4. Discussion

In this study, we generated a systematic single-cell map linking genetic risk for ten major respiratory diseases to specific lung cell types and subpopulations. Our integrative analysis reveals several disease-specific and shared susceptibility patterns across endothelial, epithelial, immune, and stromal compartments, offering mechanistic insights into how genetic risk manifests in distinct lung microenvironments. Across epithelial populations, AT0, AT1, and AT2 cells emerged as shared susceptibility types for asthma, COPD, and COVID-19, consistent with their known roles in epithelial regeneration, inflammation, and SARS-CoV-2 infection [27,29]. However, our subclustering analyses demonstrate that genetic risk is highly concentrated within discrete AT2 states. Cluster-specific enrichments—such as the innate-immune-activated AT2 cluster (cluster 2) associated with COVID-19, the apoptosis- and signal transduction AT2 cluster (cluster 4) linked to both COPD and COVID-19, and the strongly immune-regulated AT2 cluster (cluster 14) specifically associated with asthma—indicate that disease predisposition maps to distinct AT2 programs rather than to the lineage globally. These findings refine the long-standing concept of AT2 cells as progenitors by showing that genetic susceptibility preferentially targets their inflammatory, apoptotic, or repair-oriented sub-states.

Our results also highlight disease-divergent roles of specialized epithelial populations. The differential associations observed in hillock-like cells—where cluster 6 exhibits a highly proliferative, injury-responsive transcriptional program while cluster 5 shows metal-ion stress buffering features—suggest that asthma risk variants may bias hillock epithelial units toward hyper-proliferative regeneration rather than metabolic stabilization. Similarly, the separation of pre-TB secretory subclusters by their preferential associations with asthma (cluster 1) versus COVID-19 (cluster 2) indicates that subtle differences in lineage identity, rather than shared immune pathway activation, drive disease specificity. [57,58]

Within the immune compartment, the striking heterogeneity among alveolar macrophage subpopulations suggests that genetic risk is funneled through specific macrophage activation states. The COVID-19-associated CCL3^+^ macrophage subclusters showed two distinct functional programs—Group A displaying surfactant-associated and innate-immune signatures and Group B characterized by stress-response and antigen-presentation genes.

Finally, the dichotomy observed in adventitial fibroblasts highlights their dual roles as structural and immunomodulatory cells in asthma. The Group A fibroblasts—marked by antigen-presentation and NF-κB signaling—showed the strongest genetic associations, suggesting that asthma susceptibility may partly arise from fibroblast-mediated immune activation rather than remodeling alone. Conversely, the tissue-remodeling program of Group B fibroblasts implicates metabolic and extracellular-matrix pathways in chronic airway structural changes. These findings point to fibroblast heterogeneity as a central axis of asthma pathogenesis and indicate that targeting antigen-presenting fibroblast subsets could represent a previously under-recognized therapeutic avenue.

These results emphasize that genetic risk is not uniformly distributed across cell types; rather, it localizes to distinct subpopulations within lineages, underscoring the importance of single-cell resolution in understanding disease mechanisms. By linking genetic susceptibility to specific cellular states, this work provides mechanistic insights into respiratory disease heterogeneity and identifies potential cellular targets for precision therapies.

A limitation of this study is that most of the integrated GWAS datasets included in our analyses were derived predominantly from individuals of European ancestry. Given known ancestry-specific differences in allele frequencies and linkage disequilibrium patterns, the resulting cellular susceptibility profiles may not fully generalize to non-European populations. Thus, interpretations based on these datasets should be made with caution. Future studies incorporating more diverse and ancestry-balanced GWAS cohorts will be essential to improve the comprehensiveness and generalizability of cell type–disease association maps.

## 5. Conclusions

By integrating GWAS data from ten respiratory diseases with single-cell transcriptomics of the human lung, we provide a systematic map of genetic susceptibility across cellular compartments and states. We identify both shared susceptibility hubs, such as AT2 cells, and disease-specific cell types, including fibroblast and macrophage subpopulations. Importantly, our findings highlight the critical role of within-cell-type heterogeneity in mediating genetic risk. This work establishes a scalable framework for linking genetic risk to lung cellular architecture and provides mechanistic insights with implications for the development of cell-type-targeted therapies for respiratory diseases.

## Figures and Tables

**Table 1:** Summary of GWAS datasets for respiratory diseases.

Disease Name	Abbreviation	Population	Sample Size	PMID
Asthma	Asthma	European	536,345	32296059 [4]
Chronic obstructive pulmonary disease liability	COPD	European	325,027	37069358 [5]
COVID-19	COVID-19	European	1,086,211	34237774 [14]
Idiopathic fibrosing alveolitis	IFA	European	630,696	39024449 [15]
Idiopathic pulmonary fibrosis	IPF	European	1,254,748	36777996 [6]
Influenza	FLU	European	1,447,920	39103650 [16]
Lung adenocarcinoma	LUAD	European	66,756	28604730 [17]
Non-small cell lung cancer	NSCLC	European	1202	37154150 [7]
Pulmonary arterial hypertension	PAH	European	11,744	30527956 [18]
Tuberculosis	TB	European	628,203	39024449 [15]

Population indicates the primary ancestral group of the GWAS participants. For meta-analyses involving multiple populations, the major contributing population is reported. PMID refers to the PubMed ID of the corresponding GWAS publication.

## Data Availability

The GWAS summary statistics used in this study are publicly available from the GWAS Catalog (https://www.ebi.ac.uk/gwas/ (accessed on 3 July 2025)) and the Host Genetics Initiative (HGI, Release 7, https://www.covid19hg.org/results/r7/ (accessed on 3 July 2025)). The single-cell RNA sequencing data for human lung tissue were obtained from the Human Lung Cell Atlas (HLCA, https://data.humancellatlas.org/hca-bio-networks/lung/atlases/lung-v1-0 (accessed on 4 July 2025)). All datasets were accessed in accordance with the respective data usage guidelines.

## Supplementary Materials

The following supporting information can be downloaded at: https://www.mdpi.com/article/10.3390/biology14121765/s1, Figure S1: Disease correlations based on cell-type association scores; Table S1: Statistical assessment of associations and heterogeneity between respiratory diseases and lung cell types; Table S2: Proportions of cells significantly associated with disease (FDR < 0.1) within subclusters; Table S3: Functional enrichment consistency across varying up-regulated gene-number cut-offs (top 100–500) in cluster 2 of AT2 cells.

## Abbreviations

The following abbreviations are used in this manuscript:

GWAS	Genome-Wide Association Study
scRNA-seq	Single-cell RNA-sequencing
COPD	Chronic obstructive pulmonary disease liability
AT2	alveolar type II
