# Supplementary material for: Single-Cell Mapping of Genetic Risk Across Ten Respiratory Diseases
Source: Biology (Basel). 2025 Dec 10;14(12):1765. doi: 10.3390/biology14121765 (PMC12731109; doi:10.3390/biology14121765)
Supplement: Supplementary file 1 [file biology-14-01765-s001.zip › Supplementary Figure S1.pdf]

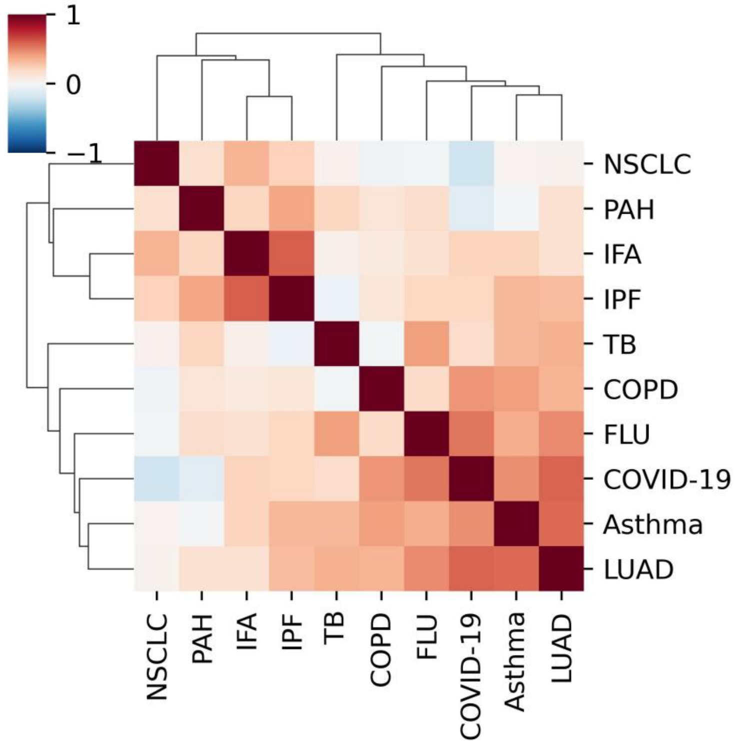

Supplementary Figure S1: Disease correlations based on cell-type association scores. Disease correlations were calculated as a Spearman correlation matrix using cell-type association scores derived from scDRS.
